# The Prince of Wales's Hospital, Cardiff

**Published:** 1921-03-12

**Authors:** 


### The Prince of Wales's Hospital, Cardiff.

DAME MARGARET LLOYD GEORGE'S WORK.

Doubtless our readers will recall that on December 4 a male voice competition was held at the Cardiff Empire and a concert at the City Hall on the evening of the same day, both of which were in aid of the Prince of Wales's Hospital for Limbless and Cripples, Cardiff. In connection with these gatherings Dame Margaret Lloyd George, Chairman of the Council of the hospital, issued a personal appeal to a number of firms and companies. The donations received since that date by Dame Margaret Lloyd George are :—

| | £ | s. | d. |
|---|---|---|---|
| Sir W. R. Smith, Bart. ... ... ... ... | £2,000 | 0 | 0 |
| Henry G. Lewis, Esq., Porthkerry, High Sheriff of Glamorgan ... ... ... | 1,000 | 0 | 0 |
| Consolidated Cambrian, Ltd. ... ... | 500 | 0 | 0 |
| United National Collieries, Ltd. ... | 210 | 0 | 0 |
| North's Navigation Collieries, Ltd. ... | 210 | 0 | 0 |
| Celtic Collieries, Ltd. ... ... ... ... | 105 | 0 | 0 |
| Sybil Viscountess Rhondda ... ... | 100 | 0 | 0 |
| Tirpentwys Black Vein Steam Coal and Coke Co., Ltd., Pontypool ... ... | 100 | 0 | 0 |
| Tredegar Iron and Coal Co., Ltd. ... | 100 | 0 | 0 |
| Blaencorrwg Colliery Co., Ltd. ... ... | 10 | 10 | 0 |
| Ebbw Vale Steel, Iron and Coal Co., Ltd. | 5 | 5 | 0 |

These are in addition to the proceeds from the competitions, etc., which were from the male voice competition at the Empire, £376, and from the concert at City Hall, £149. At the recent Executive Committee, moreover, a promise was announced from Major David Davies, M.P., and the Misses Davies of the sum of £1,000 towards the same fund.

The Hospital Endowment Fund has now reached £50,273, so that a sum of nearly £50,000 has still to be raised before the amount aimed at by Dame Margaret Lloyd George is secured.

